# Supplementary material for: Spatial readout of visual looming in the central brain of Drosophila
Source: eLife. 2020 Nov 18;9:e57685. doi: 10.7554/eLife.57685 (PMC7744102; doi:10.7554/eLife.57685)
Supplement: Supplementary file 1. [file elife-57685-supp1.docx]

**Supplementary File 1: Detailed summary of genotypes used throughout the manuscript**

| **Figure** | **Driver line(s)** | **Reporter / effector transgene(s)** | **Description (Image details for anatomy Figures)** |
| --- | --- | --- | --- |
| 1B, 1B’ | OL0070B | pJFRC51-3XUAS-IVS-syt::smHA (su(Hw)attP1) ,pJFRC225-5XUAS-IVS-myr::smFLAG (VK00005) (only myr::smFLAG pattern shown) | Composite image includes reference brain pattern  63x objective |
| 1C, 1C’ | OL0081B | MCFO-1 [pBPhsFlp2::PEST (attP3);; pJFRC201- 10XUAS-FRT>STOP>FRT-myr::smGFP-HA (VK0005), pJFRC240-10XUAS-FRT> STOP>FRT-myr::smGFP-V5-THS-10XUASFRT>STOP>FRT-myr::smGFP-FLAG (su(Hw)attP1)] | Composite image includes reference brain pattern  (63x objective) |
| 1D,1H,1I,  1J,1K,  1L,1M,  1N, 1—fs1 | OL0070B | pJFRC51-3XUAS-IVS-syt::smHA (su(Hw)attP1) ,pJFRC225-5XUAS-IVS-myr::smFLAG (VK00005) (only syt::smHA pattern shown) | LC6 glomerulus label used in several panels (manually segmented)  (63x objective) |
| 1D | OL0081B (single cell) | MCFO-1 | Composite image includes reference brain pattern and LC6 glomerulus label  (63x objective) |
| 1E | SS00825 | pJFRC51-3XUAS-IVS-syt::smHA (su(Hw)attP1) ,pJFRC225-5XUAS-IVS-myr::smFLAG (VK00005) (only myr::smFLAG pattern shown)  anti-Brp neuropile marker | Maximum intensity projection  (20x objective) |
| 1F | SS02036 | pJFRC51-3XUAS-IVS-syt::smHA (su(Hw)attP1) ,pJFRC225-5XUAS-IVS-myr::smFLAG (VK00005) (only myr::smFLAG pattern shown)  anti-Brp neuropile marker | Maximum intensity projection  (20x objective) |
| 1G | SS02036 | MCFO-1 | Composite images include reference brain pattern and LC6 glomerulus label  Manually segmented cells.  (63x objective) |
| 1H, 1—fs1A | SS00825 | MCFO-1 |  |
| 1I, 1­­—fs1B | SS00824 | MCFO-1 |  |
| 1J, 1—fs1C | SS02036 | MCFO-1 |  |
| 1K, 1—fs1D | SS02699 | MCFO-1 |  |
| 1L, 1—fs1E | SS03690 | MCFO-1 |  |
| 1M, 1—fs1F | SS02410 | MCFO-1 |  |
| 1N | NA | NA | Composite image of reference brain pattern and LC6 glomerulus label (63x objective) |
| 1O | SS03690 | pJFRC51-3XUAS-IVS-syt::smHA (su(Hw)attP1) ,pJFRC225-5XUAS-IVS-myr::smFLAG (VK00005) | Composite image with reference brain pattern  (63x objective) |
| 1P | SS02036 | pJFRC51-3XUAS-IVS-syt::smHA (su(Hw)attP1) ,pJFRC225-5XUAS-IVS-myr::smFLAG (VK00005) | Composite image with reference brain pattern  (63x objective) |
| 1Q | SS02036, SS02699, SS03690, SS02410 | MCFO-1 | Composite image with reference brain pattern  Overlay of cells from 1J, 1K, 1L, 1M (shown in a different view) |
| 2, 2—fs1: LC6 | OL0070B | FC-1 [13XLexAop2-CsChrimson-tdT (attP18), 20XUAS-IVS-Syn21-opGCaMP6f p10 Su(Hw)attp8); 42E06-LexA(JK22C);+] | Functional connectivity experiments: depolarizing LC6 with Chrimson, while imaging calcium responses from other cell type. Used with two-photon imaging. |
| 2, 2—fs1: LC6G1 | SS00825 | FC-1 |  |
| 2, 2—fs1: LC6G2 | SS02036 | FC-1 |  |
| 2, 2—fs1: LC6G3 | SS02099 | FC-1 |  |
| 2, 2—fs1: LC6G4 | SS03690 | FC-1 |  |
| 2, 2—fs1: LC6G5 | SS02409 | FC-1 |  |
| 2—fs1: LC26G1 | SS03641 | FC-1 |  |
| 3, 4, 3—fs3: LC6 | OL0070B | GCaMP-1 [pJFRC7-20XUAS-IVS-GCaMP6m (VK00005)] | Calcium imaging from either LC6 or one of the LC6G neurons. In a subset of the recordings, the LC6 glomerulus was labeled with tdTomato. Used with two-photon imaging. |
| 3, 4, 3—fs1,2: LC6G1 | SS00825 | GCaMP-1 or  GCaMP-2 [pJFRC48-13XLexAop2-IVS-myrtdTomato (su(Hw)attP8); 42E06-LexA(JK22C); pJFRC7-20XUAS-IVS-GCaMP6m (VK00005)] |  |
| 3, 4, 3—fs1,2, 8: LC6G2 | SS02036 | GCaMP-1 or GCaMP-2 |  |
| 1—fs1G, 1—fs1G’, 1—fs1H, 1—fs1H’ | SS03641 | pJFRC51-3XUAS-IVS-syt::smHA (su(Hw)attP1) ,pJFRC225-5XUAS-IVS-myr::smFLAG (VK00005) | Composite image with reference brain pattern and LC26 glomerulus label based on syt-HA expression.  Manually segmented.  (63x objective) |
| 1—fs2 | SS00825, SS02036, SS02099, SS02409,  SS03690, SS03641 | pJFRC51-3XUAS-IVS-syt::smHA (su(Hw)attP1) ,pJFRC225-5XUAS-IVS-myr::smFLAG (VK00005) (only myr::smFLAG pattern shown)  anti-Brp neuropile marker | Maximum intensity projections  (20x objective) |
| 1—fs3 | SS00825, SS02036, SS02099, SS03690, SS25111 | UAS-7xHaloTag*::*CAAX*in*VK00005  FISH probes for GAD1, VGlut, ChAT mRNAs | Single confocal sections  (63x objective) |

fs = figure supplement
