# Supplementary material for: Spatial readout of visual looming in the central brain of Drosophila
Source: eLife. 2020 Nov 18;9:e57685. doi: 10.7554/eLife.57685 (PMC7744102; doi:10.7554/eLife.57685)
Supplement: Supplementary file 2. [file elife-57685-supp2.docx]

| Stimulus type | Stimulus name | Nominal location (azimuth, elevation) | Speeds | Fig.3 | Fig.4B | Fig.4C | Fig.7B |
| --- | --- | --- | --- | --- | --- | --- | --- |
| 1 | Small looming RF mapping stimulus | 98 center positions ranging -18° to 99°, ±27° | 10°/s | small looming |  |  |  |
| 2 | Dark Looming Disc side | centered at 45°, -9° (18° for LC6G2) | r/v = 10, 40, 70, 130, 310, 550 ms |  | looming | looming 1 |  |
| 2 | Dark Receding Disc side | centered at 45°, -9° (18° for LC6G2) | r/v = 10, 40, 70, 130, 310, 550 ms |  | receding | receding 3 |  |
| 2 | Dark Looming Disc front | centered at 27°, -9° (18° for LC6G2) | r/v = 10, 40, 70, 130, 310, 550 ms |  |  | looming 4 |  |
| 2 | Dark Receding Disc front | centered at 27°, -9° (18° for LC6G2) | r/v = 10, 40, 70, 130, 310, 550 ms |  |  | receding 2 |  |
| 3 | Bright Looming Disc side | centered at 45°, -9° (18° for LC6G2) | r/v = 10, 40, 70, 130, 310, 550 ms |  | bright looming | looming 2 |  |
| 3 | Bright Receding Disc side | centered at 45°, -9° (18° for LC6G2) | r/v = 10, 40, 70, 130, 310, 550 ms |  |  | receding 1 |  |
| 4 | Dark Constant Looming Disc side | centered at 45°, -9° (18° for LC6G2) | 5,7,10,20,30,40,50,70,101,203,494°/s |  |  | looming 3 | ipsi, contra-only expanding |
| 4 | Dark Constant Receding Disc side | centered at 45°, -9° (18° for LC6G2) | 5,7,10,20,30,40,50,70,101,203,494°/s |  |  | receding 4 | ipsi-only receding |
| 5 | Luminance-matched stimulus side | centered at 45°, -9° (18° for LC6G2) | r/v = 10, 40, 70, 130, 310, 550 ms |  | luminance-matched | luminance-matched 1 |  |
| 5 | Luminance-matched receding side | centered at 45°, -9° (18° for LC6G2) | r/v = 10, 40, 70, 130, 310, 550 ms |  |  | luminance-matched 2 |  |
| 6 | Looming annulus side | centered at 45°, -9° (18° for LC6G2) | r/v = 10, 40, 70, 130, 310, 550 ms |  | looming annulus | looming annulus 1 |  |
| 6 | Looming annulus receding | centered at 45°, -9° (18° for LC6G2) | r/v = 10, 40, 70, 130, 310, 550 ms­ |  |  | looming annulus 2 |  |
| 7 | Bilateral Constant Looming Disc side | centered at 45°, 18° (for LC6G2) | 10,20,50°/s |  |  |  | ipsi-contra expanding |
| 8 | Small object motion stimulus progressive | azimuth 0° to 108°, -31.5°(31.5° for LC6G2) | 10,20°/s |  | small object | small object 1 |  |
| 8 | Small object motion stimulus regressive | azimuth 108° to 0°, -31.5°(31.5° for LC6G2) | 10,20°/s |  |  | small object 2 |  |
| 9 | Vertical bar motion stimulus progressive | azimuth 0° to 108° | 10,20°/s |  | vertical bar | vertical bar 1 |  |
| 9 | Vertical bar motion stimulus regressive | azimuth 108° to 0° | 10,20°/s |  |  | vertical bar 2 |  |

**Supplementary File 2: Detailed summary of visual stimuli**
