# Supplementary figures and images for: Spatial readout of visual looming in the central brain of Drosophila
Source: eLife. 2020 Nov 18;9:e57685. doi: 10.7554/eLife.57685 (PMC7744102; doi:10.7554/eLife.57685)

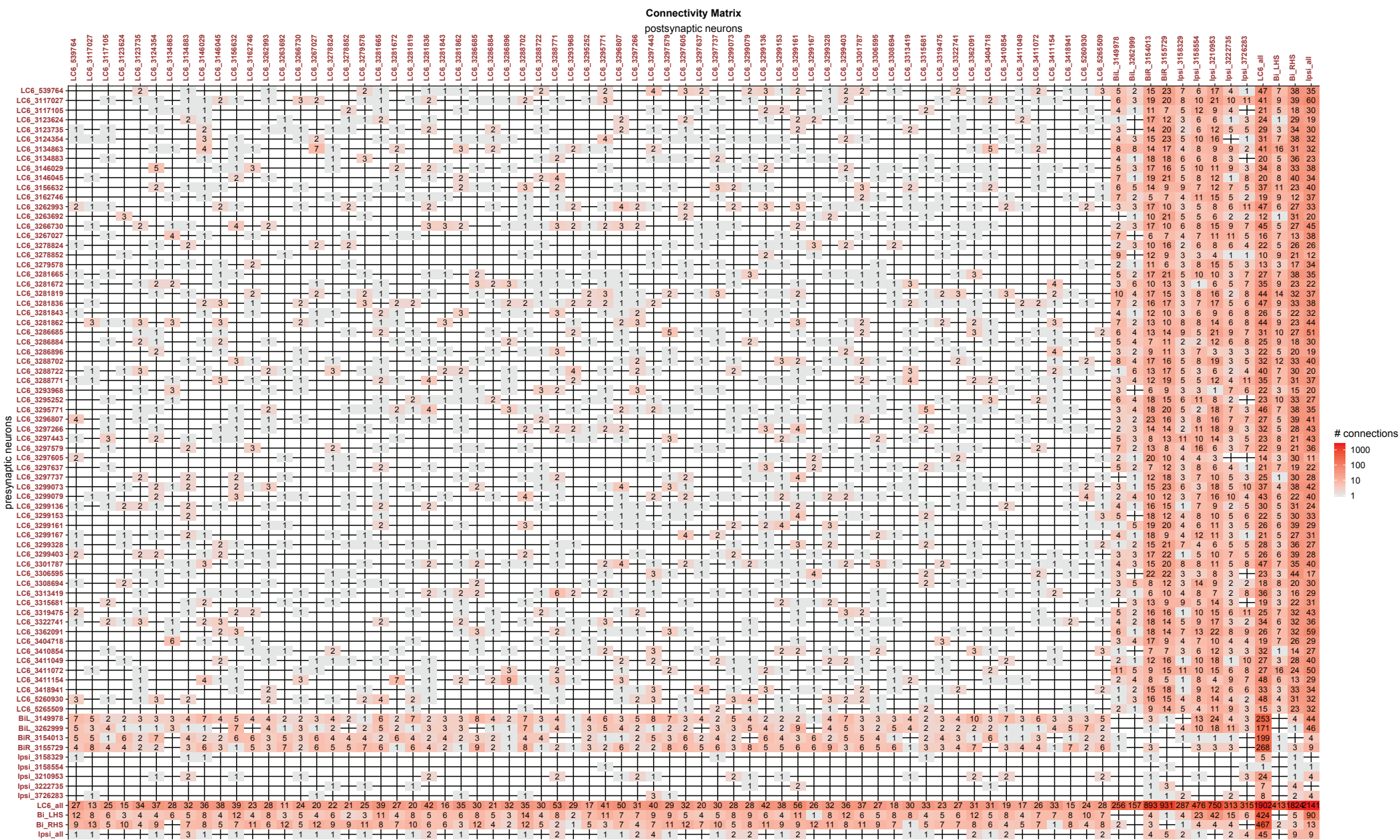

Connectivity matrix of LC6 and target neurons from EM data, related to Figure 6

Supplement: Supplementary file 3. [file elife-57685-supp3.pdf]
