# Supplementary material for: Spatial readout of visual looming in the central brain of Drosophila
Source: eLife. 2020 Nov 18;9:e57685. doi: 10.7554/eLife.57685 (PMC7744102; doi:10.7554/eLife.57685)
Supplement: Transparent reporting form [file elife-57685-transrepform.docx]

***eLife’s* transparent reporting form**

We encourage authors to provide detailed information *within their submission* to facilitate the interpretation and replication of experiments. Authors can upload supporting documentation to indicate the use of appropriate reporting guidelines for health-related research (see [EQUATOR Network](http://www.equator-network.org/%20)), life science research (see the [BioSharing Information Resource](https://biosharing.org/)), or the [ARRIVE guidelines](http://www.plosbiology.org/article/info:doi/10.1371/journal.pbio.1000412) for reporting work involving animal research. Where applicable, authors should refer to any relevant reporting standards documents in this form.

If you have any questions, please consult our Journal Policies and/or contact us: [editorial@elifesciences.org](mailto:editorial@elifesciences.org).

**Sample-size estimation**

- You should state whether an appropriate sample size was computed when the study was being designed
- You should state the statistical method of sample size computation and any required assumptions
- If no explicit power analysis was used, you should describe how you decided what sample (replicate) size (number) to use

Please outline where this information can be found within the submission (e.g., sections or figure legends), or explain why this information doesn’t apply to your submission:

No statistical methods were used to pre-determine sample sizes, however, our sample sizes are in the range of those reported in similar, previous publications. All information about statistical methods and analysis are provided in the methods section for each experiment type.

**Replicates**

- You should report how often each experiment was performed
- You should include a definition of biological versus technical replication
- The data obtained should be provided and sufficient information should be provided to indicate the number of independent biological and/or technical replicates
- If you encountered any outliers, you should describe how these were handled
- Criteria for exclusion/inclusion of data should be clearly stated
- High-throughput sequence data should be uploaded before submission, with a private link for reviewers provided (these are available from both GEO and ArrayExpress)

Please outline where this information can be found within the submission (e.g., sections or figure legends), or explain why this information doesn’t apply to your submission:

All replicate in the study are biological replicates. For the imaging experiments, the assays were performed multiple times on independent animals, with details provided in figure legends and in the methods. For the EM analysis, multiple individual neurons were reconstructed for each cell type (65, 2, 2, and 5, depending on the cell type – all detailed in figure legends and methods). For the light microscopy images, we show examples from individual samples, but have confirmed consistent results across at least 5 samples per image shown.

**Statistical reporting**

- Statistical analysis methods should be described and justified
- Raw data should be presented in figures whenever informative to do so (typically when N per group is less than 10)
- For each experiment, you should identify the statistical tests used, exact values of N, definitions of center, methods of multiple test correction, and dispersion and precision measures (e.g., mean, median, SD, SEM, confidence intervals; and, for the major substantive results, a measure of effect size (e.g., Pearson's r, Cohen's d)
- Report exact p-values wherever possible alongside the summary statistics and 95% confidence intervals. These should be reported for all key questions and not only when the p-value is less than 0.05.

Please outline where this information can be found within the submission (e.g., sections or figure legends), or explain why this information doesn’t apply to your submission:

The statistical methods are reported in each figure legend as well as in the relevant methods sections. The test used, p-value calculated, value of N, and type of confidence interval displayed are described for each relevant data panel (figure legends and related methods section).

(For large datasets, or papers with a very large number of statistical tests, you may upload a single table file with tests, Ns, etc., with reference to sections in the manuscript.)

**Group allocation**

- Indicate how samples were allocated into experimental groups (in the case of clinical studies, please specify allocation to treatment method); if randomization was used, please also state if restricted randomization was applied
- Indicate if masking was used during group allocation, data collection and/or data analysis

Please outline where this information can be found within the submission (e.g., sections or figure legends), or explain why this information doesn’t apply to your submission:

All neurons of the same type, expressed in the same driver line, were allocated to a group. No further group selection was performed.

**Additional data files (“source data”)**

- We encourage you to upload relevant additional data files, such as numerical data that are represented as a graph in a figure, or as a summary table
- Where provided, these should be in the most useful format, and they can be uploaded as “Source data” files linked to a main figure or table
- Include model definition files including the full list of parameters used
- Include code used for data analysis (e.g., R, MatLab)
- Avoid stating that data files are “available upon request”

Please indicate the figures or tables for which source data files have been provided:

An accompanying code and data repository is already publicly available: <https://github.com/reiserlab/LC6downstream>. Due to very large data set size, it is impractical to share all raw calcium imaging data, but the primary summary data is available in this repository (for Figures 3, 5, and 6). All reconstructed neurons described in the manuscript will be shortly available at <https://fafb.catmaid.virtualflybrain.org/>, and all anatomy analysis code is available in the repository listed above.
